# Novel Topologically Complex Scaffold Derived from Alkaloid Haemanthamine

**DOI:** 10.3390/molecules23020255

**Published:** 2018-01-28

**Authors:** Karthik Govindaraju, Marco Masi, Margaux Colin, Veronique Mathieu, Antonio Evidente, Todd W. Hudnall, Alexander Kornienko

**Affiliations:** 1 Department of Chemistry and Biochemistry, Texas State University, San Marcos, TX 78666, USA; gkiict@gmail.com; 2 Dipartimento di Scienze Chimiche, Università di Napoli Federico II, Complesso Universitario Monte Sant’Angelo, Via Cintia 4, 80126 Napoli, Italy; marco.masi@unina.it (M.M.); evidente@unina.it (A.E.); 3 Department of Pharmacotherapy and Pharmaceutics, Faculté de Pharmacie, Université Libre de Bruxelles (ULB), 1050 Brussels, Belgium; macolin@ulb.ac.be (M.C.); veronique.mathieu@ulb.ac.be (V.M.)

**Keywords:** natural product-like, diversity-oriented synthesis, haemanthamine, haemanthidine, iodoalkoxylation, biological activity

## Abstract

The generation of natural product-like compound collections has become an important area of research due to low hit rates found with synthetic high-throughput libraries. One method of generating compounds occupying the areas of chemical space not accessible to synthetic planar heterocyclic structures is the utilization of natural products as starting materials. In the current work, using a ring-closing iodoalkoxylation reaction, alkaloid haemanthamine was transformed into a unique structural framework possessing an intricate ring system and a large number of stereocenters. The structure of the new compound was confirmed with an X-ray analysis. A small number of derivatives of this new compound were synthesized as a demonstration of the possibility of generating a large natural product-like compound collection based on the new structural framework.

## 1. Introduction

Although the libraries of small molecules utilized in drug discovery are still dominated by planar compounds with high sp^2^ character and low stereochemical complexity, the low hit rates obtained with such compounds in conventional high-throughput screening campaigns have spurred many creative strategies to diversify screening libraries with natural product-like compounds [1]. An important approach to achieving this goal is diversity-oriented synthesis involving the use of simple starting materials to generate compounds with complex structures, incorporating intricate ring systems and large numbers of stereogenic centers [2,3,4,5]. Another method of generating compounds occupying the areas of chemical space not accessible to planar heterocyclic structures is the utilization of natural products as starting materials. This involves derivatization by interconverting functional groups [6,7] or altering a core framework of the starting natural products [8,9,10,11]. The latter approach is less common, although a number of reports disclosing creative natural product “remodeling” transformations have appeared in the literature. Some early examples include transformations of macrocyclic lathyrane diterpenes into a variety of polycyclic structures using transannular reactions [12], utilization of an elimination-[2+2] reaction scheme to elaborate sesquiterpene *R*-(+)-sclareolide into several diverse scaffolds [13], and elaboration of sesquiterpene zerumbone into more complex natural product-like ring systems with significant structural differences [14]. In an impressive, more recent, example, a ring-distortion strategy was applied to diversify the structures of gibberellic acid, adrenosterone and quinine through a large variety of skeletal rearrangements, involving ring cleavage, ring expansion, ring fusion, ring rearrangement and various combinations thereof [15]. 

## 2. Results and Discussion

**Figure 1:**
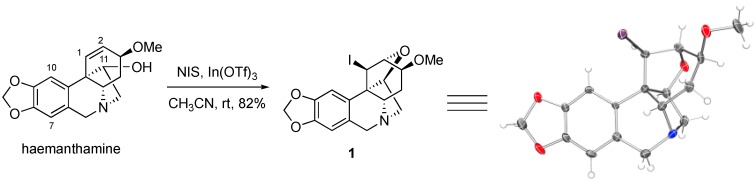
Synthesis of compound **1** and its x-ray structure. Thermal elipsoids are drawn at the 50% probability level.

The alkaloid haemanthamine (see Figure 1 for structure) has attracted interest due to its promising anticancer activities, specifically its ability to overcome cancer cell resistance to initiate apoptotic cell death, as was reported by our collaborative team [16]. This natural product has undergone a thorough chemical derivatization study, as reported by Estéves-Braun and coworkers [10]. Specifically, haemanthamine was subjected to acylation at the C11-OH (see Figure 1 for positon numbering), oxidation of the C11-OH to a ketone and subsequently conversion to an oxime, oxidation of the tertiary nitrogen to an *N*-oxide, removal of the methyl group from the C3-MeO and methylene from the C8,C9-methylenedioxy moiety, various derivatizations and reduction of the C1,C2-alkene, among several other functional group interconversions [17]. Despite the significant number of compounds prepared, the aromatic positions C7 and C10 were not engaged. During our own synthetic work with haemanthamine, we thus attempted to take advantage of the electron-rich character of these aromatic sites and iodinate at the C7 and/or C10 positions with *N*-iodosuccinimide in the presence of indium (III) triflate, following a procedure reported by Romo and coworkers [18]. To our surprise, instead of aromatic iodination, these reaction conditions led to the unexpected generation of C1,C2-iodoalkoxylated compound **1**, whose structure was confirmed with an X-ray analysis of the crystals grown in CH_3_OH/CHCl_3_.

Compound **1** represents a unique structural framework with 9 individual rings and 6 stereogenic centers, which highlights its high topological and stereochemical complexity, despite its small molecular size. It is of interest that in their report, Estéves-Braun and coworkers treated haemanthamine with electrophilic halogenating agents, such as Br_2_ and CH_3_CONHBr/SnCl_4_/H_2_O, but only isolated the C1,C2-dibromo compound in the former case and a bromohydrin in the latter [17]. It appears that in our case, the absence of a strong nucleophile leads to the intramolecular nucleophilic attack by C11-OH onto the iodonium ion at C1,C2 despite the strain associated with such dense intricate ring system. It is also of interest that the alkaloid haemanthamine has been previously reported to undergo a different type of rearrangement leading to the alkaloid montanine skeleton utilizing the activation of the C11-hydroxyl [19,20]. This illustrates the rich chemistry that haemanthamine can participate in and provides additional impetus for its synthetic studies.

**Figure 2:**
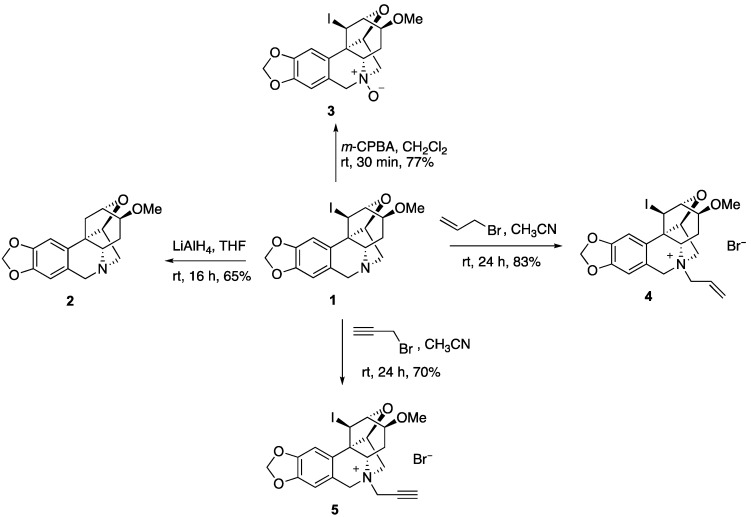
Derivatization of compound **1.**

To utilize compound **1** as a starting point to generate a collection of compounds with high topological and stereochemical diversity, we conducted a preliminary exploration of its chemistry (Figure 2). Thus, the iodine can be removed with LiAlH_4_ to generate deiodo compound **2**. Unfortunately, all attempts to utilize the secondary iodine for transition metal coupling chemistry failed and resulted in ring opening to regenerate the starting alkaloid. The tertiary nitrogen in **1** can be oxidized with *m*-CPBA to yield *N*-oxide **3** or alkylated to afford *N*-allyl and *N*-propargyl derivatives **4** and **5**.

**Figure 3:**
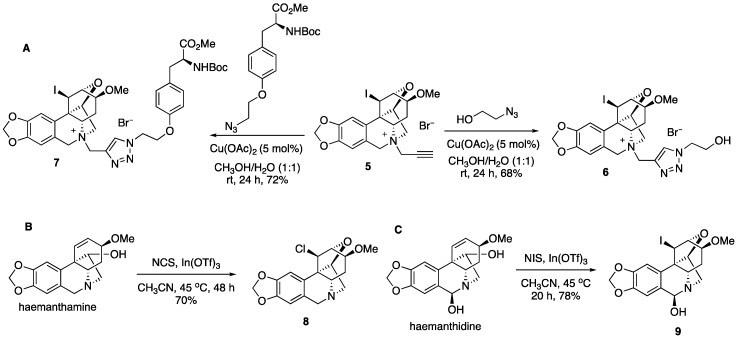
(**A**) Click reactions of compound **5**, (**B**) chloro variant of the original transformation, and (**C**) an analogous transformation of alkaloid haemanthidine.

The installation of the propargyl group in **5** allows for click chemistry to further diversify this collection of compounds (Figure 3). Thus, the coupling reaction with azido ethanol gives triazole **6**, while the azide derived from the protected tyrosine amino acid leads to complex derivative **7**. We also found that after prolonged heating at 45 °C in the presence of *N*-chlorosuccinimide, a similar transformation leads to chloro derivative **8**. Finally, the related alkaloid haemanthidine, containing a C6-hydroxyl is also capable of similar chemistry yielding compound **9** after the treatment with *N*-iodosuccinimide in the presence of indium (III) triflate. 

## 3. Materials and Methods

### 3.1. General Information

All reagents, solvents and catalysts were purchased from commercial sources (Acros Organics; Sigma-Aldrich, St. Louis, MO, USA) and used without purification. All reactions were performed in oven-dried flasks open to the atmosphere or under nitrogen and monitored by thin layer chromatography (TLC) on TLC precoated (250 μm) silica gel 60 F254 glass-backed plates (EMD Chemicals Inc., Iselin, NJ, USA). Visualization was accomplished with UV light. Flash column chromatography was performed on silica gel (32-63 μm, 60 Å pore size). ^1^H and ^13^C NMR spectra were recorded on Bruker 400 spectrometer. Chemical shifts (δ) are reported in ppm relative to the TMS internal standard. HRMS analyses were performed using Waters Synapt G2 LCMS. Haemanthamine was isolated from *Narcissus pseudonarcissus* var. King Alfred bulbs as reported previously [21]. Haemanthidine was purified from the dried bulbs of *Lycoris aurea* Herb. as reported previously [16]. 

### 3.2. Crystallographic Information

The crystallographic measurement of compound **1** was carried out on a Rigaku Mini CCD area detector diffractometer using graphite-monochromated Mo Kα radiation (λ = 0.71075 Å) at 150(2) K using an Oxford Cryostream low-temperature device. A sample of suitable size and quality was selected and mounted onto a nylon loop. Data reductions were performed using Crystal Clear Expert 2.0. The structures were solved by direct methods, which successfully located most of the non-hydrogen atoms. Subsequent refinements on *F^2^* using the SHELXTL/PC package (version 5.1) allowed location of the remaining non-hydrogen atoms. Crystal Data for **1**, C_17_H_18_INO_4_ (M = 427.22 g/mol): orthorhombic, space group P2_1_2_1_2_1_, a = 7.7167(11) Å, b = 12.2406(17) Å, c = 16.908(3) Å, α = 90.0°, β = 90.0°, γ = 90.0°, V = 1597.1(4) Å^3^, Z = 4, T = 150(2) K, μ(MoKα) = 0.71075 mm^-1^, Dcalc = 1.777 g/cm^3^, 13403 reflections measured (3.121° ≤ 2Θ ≤ 24.998°), 2808 unique (Rint = 0.0563, Rsigma = 0.0429), which were used in all calculations. The final R1 was 0.0443 (I > 2σ(I)) and wR2 was 0.0795 (all data), and the goodness of fit (GooF) on *F^2^* was 1.004. The corresponding crystallographic information file (.cif file) was deposited into the Cambridge Crystallographic Data Centre (CCDC) and was assigned the following reference number: 1813576. These data can be obtained free of charge via http://www.ccdc.cam.ac.uk/conts/retrieving.html (or from the CCDC, 12 Union Road, Cambridge CB2 1EZ, UK; Fax: +44 1223 336033; E-mail: deposit@ccdc.cam.ac.uk).

### 3.3. Experimental Procedures

13-*Iodo*-4-*methoxy*-1,3,4,5,5a,7-*hexahydro*-1,6:3,12b-*dimethano*[1,3]*dioxolo*[4,5-g]*oxepino*[4,3-c]*isoquinoline* (**1**). To a solution of haemanthamine (100 mg, 0.332 mmol) in dry CH_3_CN (5 mL) was added In(OTf)_3_ (93 mg, 0.166 mmol) at room temperature under N_2_ gas. After stirring for 30 min, to the solution was added NIS (89 mg, 0.4 mmol) and the reaction mixture was stirred at 45 °C for 2 h. The solvent was removed under reduced pressure and the resulting residue was purified by preparative TLC using CH_2_Cl_2_/MeOH (9.5:0.5) as an eluent to yield 116 mg of **1** (82%) as an amorphous white solid. ^1^H NMR (400 MHz, CDCl_3_) δ 6.63 (s, 1H), 6.49 (s, 1H), 5.93 (d, *J* = 2.8 Hz, 2H), 4.48 (d, *J* = 4.9 Hz, 1H), 4.46–4.36 (m, 2H), 4.14 (dd, *J* = 6.3, 2.9 Hz, 1H), 3.78–3.62 (m, 2H), 3.61–3.52 (m, 1H), 3.46–3.36 (m, 5H), 3.17 (d, *J* = 14.2 Hz, 1H), 2.51 (ddd, *J* = 16.7, 12.5, 7.2 Hz, 1H), 2.06 (dd, *J* = 15.7, 9.5 Hz, 1H); ^13^C NMR (100 MHz, CDCl_3_) δ 146.8, 146.5, 131.0, 127.2, 106.7, 102.1, 101.0, 87.3, 80.0, 77.8, 64.3, 64.2, 60.7, 57.0, 55.7, 30.3, 14.3; HRMS (ESI) calcd for C_17_H_19_INO_4_^+^, 428.0359 (M+H)^+^; Found, 428.0362. (Supplementary Materials NMR Spectra)

4-*Methoxy*-1,3,4,5,5a,7-*hexahydro*-1,6:3,12b-*dimethano*[1,3]*dioxolo*[4,5-g]*oxepino*[4,3-c]*isoquinoline* (**2**). To a solution of **1** (30 mg, 0.071 mmol) in dry THF (3 mL) was added LiAlH_4_ (8 mg, 0.11 mmol) at 0 °C under N_2_ gas and the reaction mixture was stirred at rt for 16 h. Ice water was added, and the mixture was filtered and extracted with CH_2_Cl_2_. The organic phase was dried over anhydrous Na_2_SO_4_, filtered and concentrated. The resulting residue was purified by preparative TLC using CH_2_Cl_2_/MeOH (9.5:0.5) as an eluent to yield 14 mg of **2** (65%) as an amorphous white solid. ^1^H NMR (400 MHz, CDCl_3_) δ 6.75 (s, 1H), 6.50 (s, 1H), 5.95–5.90 (m, 2H), 4.56–4.44 (m, 2H), 4.27 (d, *J* = 9.5 Hz, 1H), 3.96–3.80 (m, 1H), 3.77–3.45 (m, 4H), 3.36–3.30 (m, 3H), 3.30–3.19 (m, 1H), 2.48 (dd, *J* = 12.0, 6.5 Hz, 1H), 2.31 (dd, *J* = 19.0, 11.4 Hz, 1H), 2.06–1.93 (m, 1H); HRMS (ESI) calcd for C_17_H_20_NO_4_^+^, 302.1392 (M+H)^+^; Found, 302.1406.

13-*Iodo*-4-*methoxy*-3,4,5,5a,6,7-*hexahydro*-1H-1,6:3,12b-*dimethano*[1,3]*dioxolo*[4,5-g]*oxepino*[4,3-c]*isoquinoline* 6-*oxide* (**3**). To a solution of **1** (20 mg, 0.047 mmol) in dry CH_2_Cl_2_ (2 mL) was added *m*-CPBA (8.1 mg, 0.047 mmol) at room temperature under N_2_ gas and the reaction mixture was stirred for 30 min. The solvent was removed under reduced pressure. The resulting residue was purified by preparative TLC using CH_2_Cl_2_/MeOH (8:2) as an eluent to yield 15.9 mg of **3** (77%) as an amorphous white solid. ^1^H NMR (400 MHz, CDCl_3_) δ 6.64 (s, 1H), 6.62 (s, 1H), 6.04–5.98 (m, 2H), 4.98–4.88 (m, 2H), 4.53 (d, *J* = 5.0 Hz, 1H), 4.40 (d, *J* = 5.0 Hz, 1H), 4.30 (dd, *J* = 13.2, 6.3 Hz, 1H), 4.05 (dd, *J* = 6.2, 3.0 Hz, 1H), 3.83 (d, *J* = 10.1 Hz, 1H), 3.69 (dd, *J* = 20.8, 14.6 Hz, 3H), 3.52–3.42 (m, 3H), 2.27 (ddd, *J* = 16.1, 10.2, 5.7 Hz, 1H).

6-*Allyl*-13-*iodo*-4-*methoxy*-3,4,5,5a,6,7-*hexahydro*-1H-1,6:3,12b-*dimethano*[1,3]*dioxolo*[4,5-g]*oxepino*[4,3-c]*isoquinolin*-6-*ium* (**4**). To a solution of **1** (20 mg, 0.047 mmol) in dry CH_3_CN (3 mL) was added allyl bromide (12 μL, 0.141 mmol) under N_2_ gas and the reaction mixture was stirred at room temperature for 24 h. The solvent was removed under reduced pressure. The resulting residue was purified by preparative TLC using CH_2_Cl_2_/MeOH (8:2) as an eluent to yield 18.2 mg of **4** (83%) as a semi solid. ^1^H NMR (400 MHz, DMSO) δ 6.92 (d, *J* = 10.9 Hz, 2H), 6.07 (s, 2H), 5.68 (dd, *J* = 24.4, 13.5 Hz, 2H), 4.88 (dd, *J* = 38.0, 10.3 Hz, 2H), 4.65–4.30 (m, 6H), 4.23–4.15 (m, 1H), 3.87–3.65 (m, 2H), 3.51 (s, 1H), 3.39 (s, 3H), 2.76 (dd, *J* = 17.2, 8.7 Hz, 1H), 2.43–2.32 (m, 1H); ^13^C NMR (100 MHz, DMSO) δ 147.3, 147.1, 127.2, 126.9, 126.2, 121.3, 106.5, 102.9, 101.6, 82.6, 77.6, 77.3, 71.5, 69.8, 69.4, 65.5, 62.5, 56.6, 55.9, 21.3, 11.4; HRMS (ESI) calcd for C_20_H_23_INO_4_^+^, 468.0672 M ^+^; Found, 468.0691.

13-*Iodo*-4-*methoxy*-6-(*prop*-2-*yn*-*1*-*yl*)-3,4,5,5a,6,7-*hexahydro*-1*H*-1,6:3,12b-*dimethano*[1,3]*dioxolo*[4,5-g]*oxepino*[4,3-c]*isoquinolin*-6-*ium* (**5**). To a solution of **1** (20 mg, 0.047 mmol) in dry CH_3_CN (3 mL) was added propargyl bromide (11 μL, 0.14 mmol) under N_2_ gas and the reaction mixture was stirred at room temperature for 24 h. The solvent was removed under reduced pressure and the resulting residue was purified by preparative TLC using CH_2_Cl_2_/MeOH (4:1) as an eluent to yield 15.3 mg of **5** (70%) as an amorphous white solid. ^1^H NMR (400 MHz, DMSO) δ 6.99 (s, 1H), 6.94 (s, 1H), 6.08 (s, 2H), 5.03 (d, *J* = 17.3 Hz, 1H), 5.00–4.78 (m, 4H), 4.51 (dd, *J* = 14.6, 5.8 Hz, 2H), 4.32 (d, *J* = 9.6 Hz, 1H), 4.23 (dd, *J* = 6.4, 2.7 Hz, 1H), 4.13 (t, *J* = 2.3 Hz, 1H), 3.88–3.69 (m, 2H), 3.39 (s, 3H), 2.70 (dd, *J* = 11.8, 7.6 Hz, 1H), 2.47–2.28 (m, 1H); ^13^C NMR (100 MHz, DMSO) δ 147.4, 147.2, 127.1, 121.0, 106.7, 102.9, 101.7, 83.3, 82.8, 79.2, 77.7, 77.3, 72.4, 71.9, 69.4, 66.5, 56.6, 55.9, 50.3, 21.7, 11.2; HRMS (ESI) calcd for C_20_H_21_INO_4_^+^, 466.0515 M ^+^; Found, 466.0536.

6-((1-(2-*Hydroxyethyl*)-1*H*-1,2,3*-triazol*-4-*yl*)*methyl*)-13-*iodo*-4-*methoxy*-3,4,5,5a,6,7-*hexahydro*-1*H*-1,6:3,12b-*dimethano*[1,3]*dioxolo*[4,5-g]*oxepino*[4,3-c]*isoquinolin*-6-*ium* (**6**). To a solution of **5** (10 mg, 0.021 mmol) and 2-azidoethanol (12 mg, 0.063 mmol) in methanol/water (1:1) was added Cu(OAc)_2_ (2 mg, 0.01 mmol) and the reaction mixture was stirred at room temperature for 24 h. The reaction mixture was filtered and the solvent was removed under reduced pressure. The resulting residue was purified by preparative TLC using CH_2_Cl_2_/MeOH (4:1) as an eluent to yield 8.1 mg of **6** (68%) as an amorphous white solid. ^1^H NMR (400 MHz, DMSO) δ 8.45 (s, 1H), 6.93 (s, 1H), 6.86 (s, 1H), 6.06 (s, 2H), 5.29–4.90 (m, 5H), 4.64–4.15 (m, 6H), 4.01 (d, *J* = 13.4 Hz, 1H), 3.91–3.76 (m, 3H), 3.44 (s, 3H), 3.09 (dd, *J* = 17.2, 8.6 Hz, 1H), 2.48–2.38 (m, 1H), 1.24 (s, 1H); ^13^C NMR (100 MHz, DMSO) δ 147.3, 147.1, 134.9, 128.2, 127.1, 121.2, 106.6, 102.8, 101.6, 82.5, 77.6, 77.4, 71.6, 65.3, 59.7, 56.7, 56.0, 52.6, 21.9, 11.5. HRMS (ESI) calcd for C_22_H_26_IN_4_O_5_^+^, 553.0948 M ^+^; Found, 553.0942.

6-((1-(2-(4-((*S*)-2-((*tert*-*Butoxycarbonyl*)*amino*)-3-*methoxy*-3-*oxopropy*l)*phenoxy*)*ethyl*)-1*H*-1,2,3-*triazol*-4-*yl*)*methyl*)-13-*iodo*-4-*methoxy*-3,4,5,5a,6,7-*hexahydro*-1*H*-1,6:3,12b-*dimethano*[1,3]*dioxolo*[4,5-g]*oxepino*[4,3-c]*isoquinolin*-6-*ium* (**7**). To a solution of **5** (10 mg, 0.021 mmol) and (*S*)-methyl 3-(4-(2-azidoethoxy)phenyl)-2-((*tert*-butoxycarbonyl)amino)propanoate (10 mg, 0.025 mmol) in methanol/water (1:1) was added Cu(OAc)_2_ (5 mol%) and the reaction mixture was stirred at room temperature for 24 h. The solvent was filtered and removed under reduced pressure. The resulting residue was purified by preparative TLC using CH_2_Cl_2_/MeOH (4:1) as an eluent to yield 12.8 mg of **7** (72%) as an amorphous white solid. ^1^H NMR (400 MHz, DMSO) δ 8.56 (d, *J* = 4.9 Hz, 1H), 7.23 (d, *J* = 8.1 Hz, 1H), 7.12 (d, *J* = 8.3 Hz, 2H), 6.91 (s, 1H), 6.88–6.79 (m, 2H), 6.07 (s, 2H), 5.31–5.04 (m, 2H), 5.01–4.72 (m, 4H), 4.51 (d, *J* = 21.8 Hz, 1H), 4.41 (dd, *J* = 10.4, 5.2 Hz, 3H), 4.35–4.15 (m, 3H), 4.17–3.92 (m, 2H), 3.84 (t, *J* = 8.3 Hz, 1H), 3.68–3.53 (m, 3H), 3.41 (s, 3H), 3.07 (dd, *J* = 17.5, 8.5 Hz, 1H), 2.93–2.69 (m, 3H), 2.47–2.32 (m, 1H), 1.32 (s, 9H); ^13^C NMR (100 MHz, DMSO) δ 172.6, 156.5, 155.4, 147.3, 147.1, 135.2, 130.2, 128.4, 127.1, 121.1, 114.4, 106.5, 102.8, 101.6, 82.5, 78.3, 77.6, 77.4, 71.7, 69.6, 66.0, 65.4, 59.5, 56.6, 56.0, 55.4, 54.4, 51.7, 49.4, 41.1, 35.5, 31.3, 30.7, 28.1, 21.9, 21.0, 11.5; HRMS (ESI) calcd for C_37_H_45_IN_5_O_9_^+^, 830.2262 M ^+^; Found, 830.2290.

13-*Chloro*-4-*methoxy*-1,3,4,5,5a,7-*hexahydro*-1,6:3,12b-*dimethano*[1,3]*dioxolo*[4,5-g]*oxepino*[4,3-c]*isoquinoline* (**8**). To a solution of haemanthamine (30 mg, 0.1 mmol) in dry CH_3_CN (3 mL) was added In(OTf)_3_ (28 mg, 0.05 mmol) at rt under N_2_ gas. After stirring for 30 min, to the solution was added NCS (16 mg, 0.12 mmol) and the reaction mixture was stirred at 45 °C for 2 h. The solvent was removed under reduced pressure. The resulting residue was purified by preparative TLC using CH_2_Cl_2_/MeOH (9.5:0.5) as an eluent to yield 23.4 mg of **3** (70%) as a semi solid. ^1^H NMR (400 MHz, CDCl_3_) δ 6.88 (s, 1H), 6.57 (s, 1H), 6.01–5.96 (m, 2H), 4.74–4.46 (m, 2H), 4.09 (d, *J* = 17.4 Hz, 2H), 3.94–3.80 (m, 1H), 3.68 (dd, *J* = 20.7, 12.0 Hz, 2H), 3.40 (ddd, *J* = 17.9, 14.3, 2.5 Hz, 2H), 2.75–2.68 (m, 3H), 2.38 (dd, *J* = 15.2, 2.5 Hz, 1H), 1.95 (dd, *J* = 15.1, 10.1 Hz, 1H).

13-*Iodo*-4-*methoxy*-1,3,4,5,5a,7-*hexahydro*-1,6:3,12b-*dimethano*[1,3]*dioxolo*[4,5-g]*oxepino*[4,3-c]*isoquinolin*-7-*ol* (**9**). To a solution of haemanthidine (15 mg, 0.047 mmol) in dry CH_3_CN (3.5 mL) was added In(OTf)_3_ (14 mg, 0.0235 mmol) at rt under N_2_ gas. After stirring for 30 min, to the solution was added NIS (12.8 mg, 0.055 mmol) and the reaction mixture was stirred at 45 °C for 2 h. The solvent was removed under reduced pressure. The resulting residue was purified by preparative TLC using CH_2_Cl_2_/MeOH (9.5:0.5) as an eluent to yield 16.38 mg of **9** (78%) as an amorphous white solid. ^1^H NMR (400 MHz, CDCl_3_) δ 6.89 (s, 1H), 6.63 (s, 1H), 5.99 (ddd, *J* = 4.3, 3.2, 1.4 Hz, 2H), 5.05 (s, 1H), 4.46 (dt, *J* = 5.2, 3.4 Hz, 2H), 4.08 (dd, *J* = 6.3, 2.9 Hz, 1H), 3.85 (d, *J* = 10.7 Hz, 1H), 3.74–3.56 (m, 3H), 3.44 (s, 3H), 3.16 (dd, *J* = 14.4, 3.1 Hz, 1H), 2.64–2.49 (m, 1H), 2.09–1.94 (m, 1H); HRMS (ESI) calcd for C_17_H_19_INO_5_^+^, 444.0308 (M+H)^+^; Found, 444.0323.

## 4. Conclusions

In conclusion, we showed that a topologically and stereochemically more complex molecular framework is accessible from the alkaloid haemathamine. The preliminary synthetic studies also showed that a collection of compounds based on this framework can be readily accessed. The described chemistry, as well as many other possibilities, can lead to the generation of a library of natural product-like compounds with this unique intricate ring system for biological testing. The initial biological testing of the synthesized compounds has revealed weak antiproliferative properties associated with some of them (data not shown); however, more testing for various types of activities is underway. The described work emphasizes the utility of natural products as starting materials to generate highly structurally complex molecules.

## Supplementary Materials

Supplementary materials are available online, copies of NMR spectra.
