# Supplementary material for: Novel Topologically Complex Scaffold Derived from Alkaloid Haemanthamine
Source: Molecules. 2018 Jan 28;23(2):255. doi: 10.3390/molecules23020255 (PMC6017887; doi:10.3390/molecules23020255)

Supporting Information

Novel Topologically Complex Scaffold Derived from Alkaloid Haemanthamine

Karthik Govindaraju [1], Marco Masi [2], Margaux Colin[3], Veronique Mathieu[3], Antonio Evidente[2], Todd W. Hudnall [1,*] and Alexander Kornienko [1,*]

[1] Department of Chemistry and Biochemistry, Texas State University, San Marcos, TX 78666
[2] Dipartimento di Scienze Chimiche, Università di Napoli Federico II, Complesso Universitario Monte Sant'Angelo, Via Cintia 4, 80126, Napoli, Italy
[3] Department of Pharmacotherapy and Pharmaceutics, Faculté de Pharmacie, Université Libre de Bruxelles (ULB), Brussels, Belgium

NMR Spectra

1: ^{1}H NMR (400 MHz, $CDCl_3$)

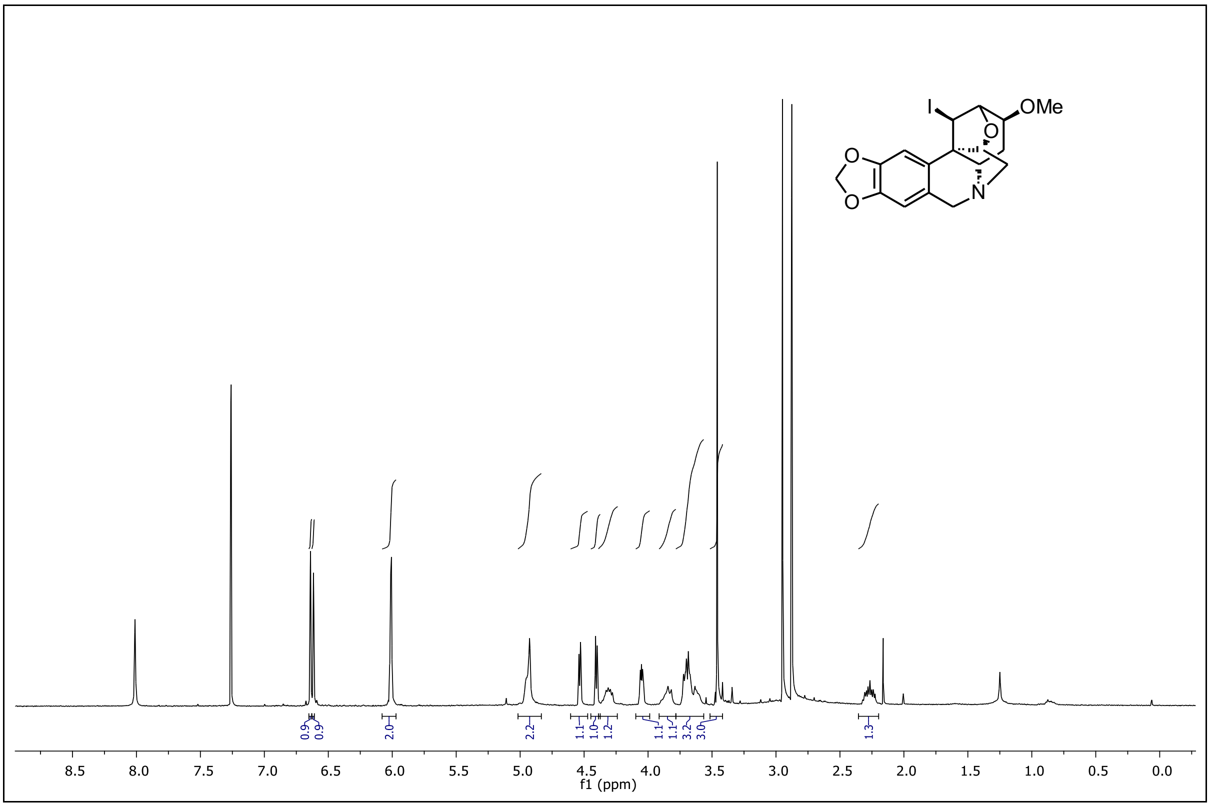

1: ^{13}C NMR (100 MHz, $CDCl_3$)

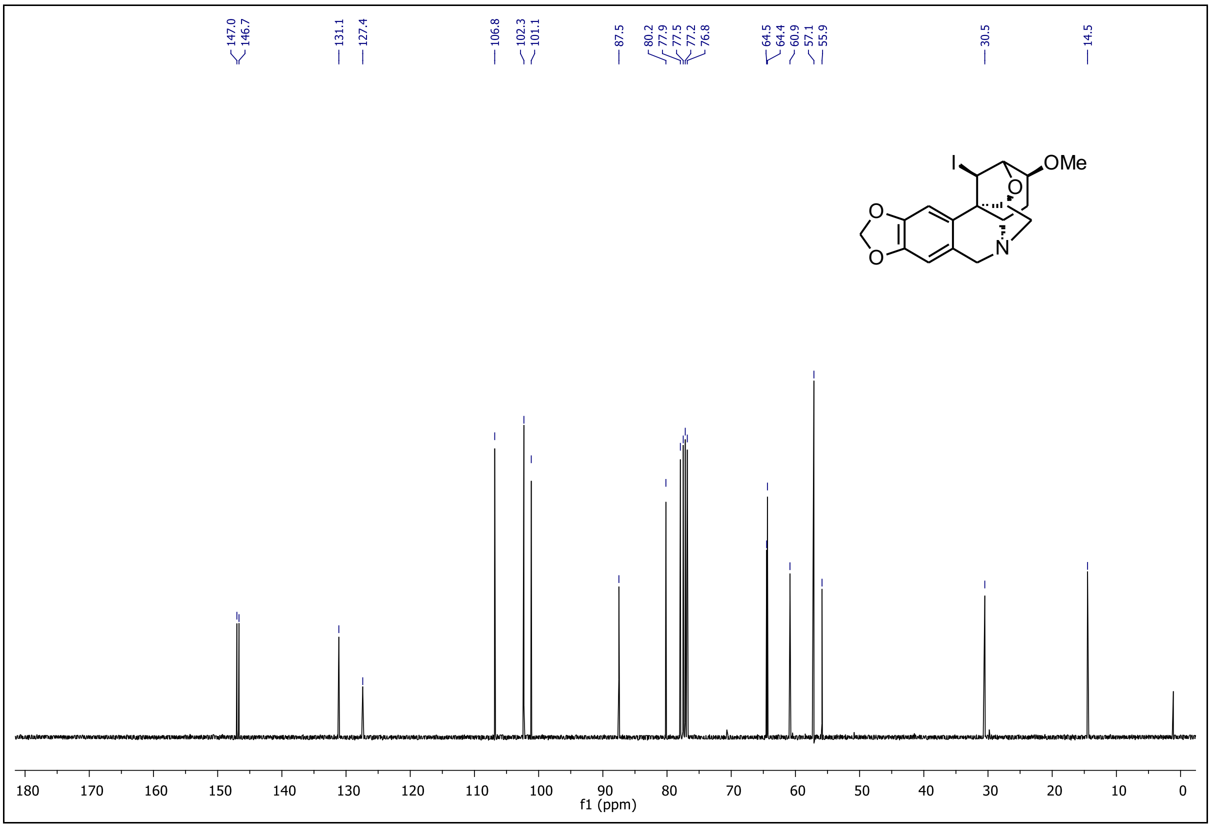

2: ^{1}H NMR (400 MHz, $CDCl_3$)

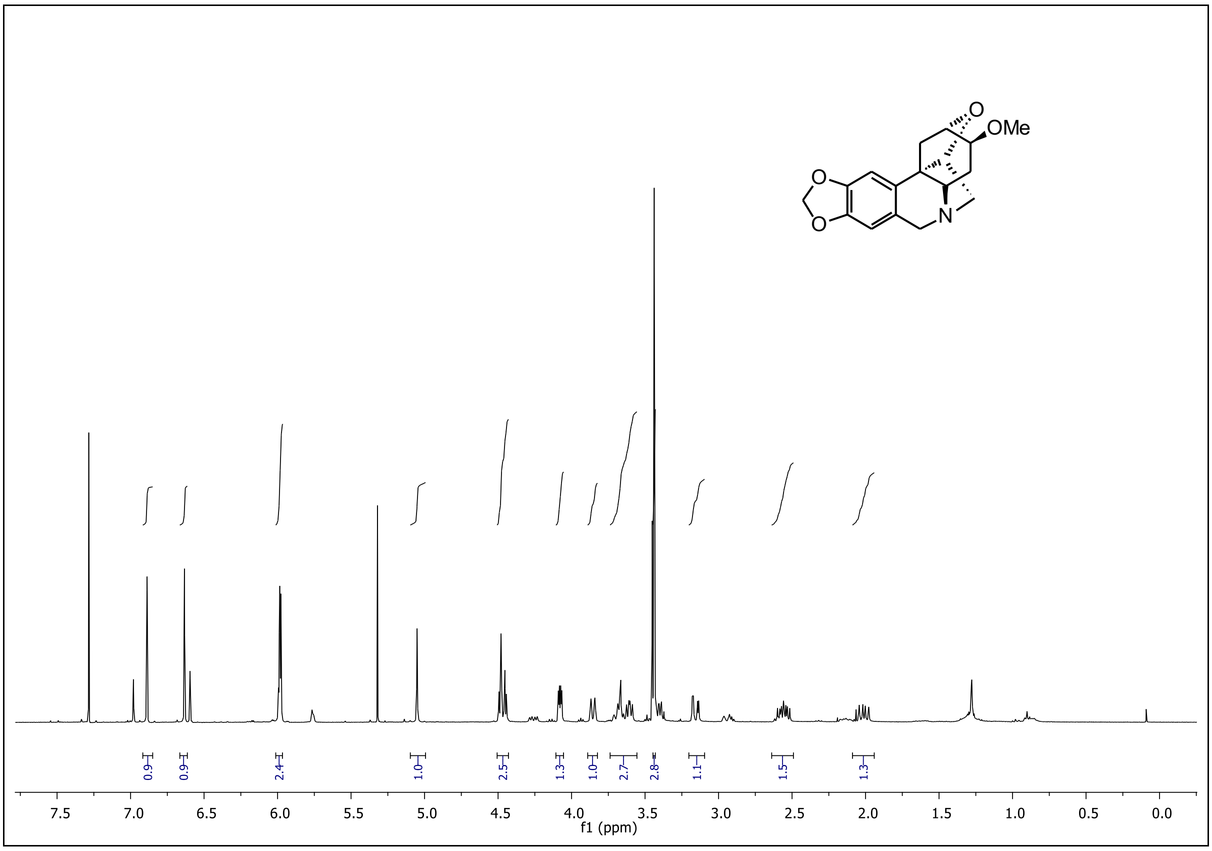

3: ^{1}H NMR (400 MHz, $CDCl_3$)

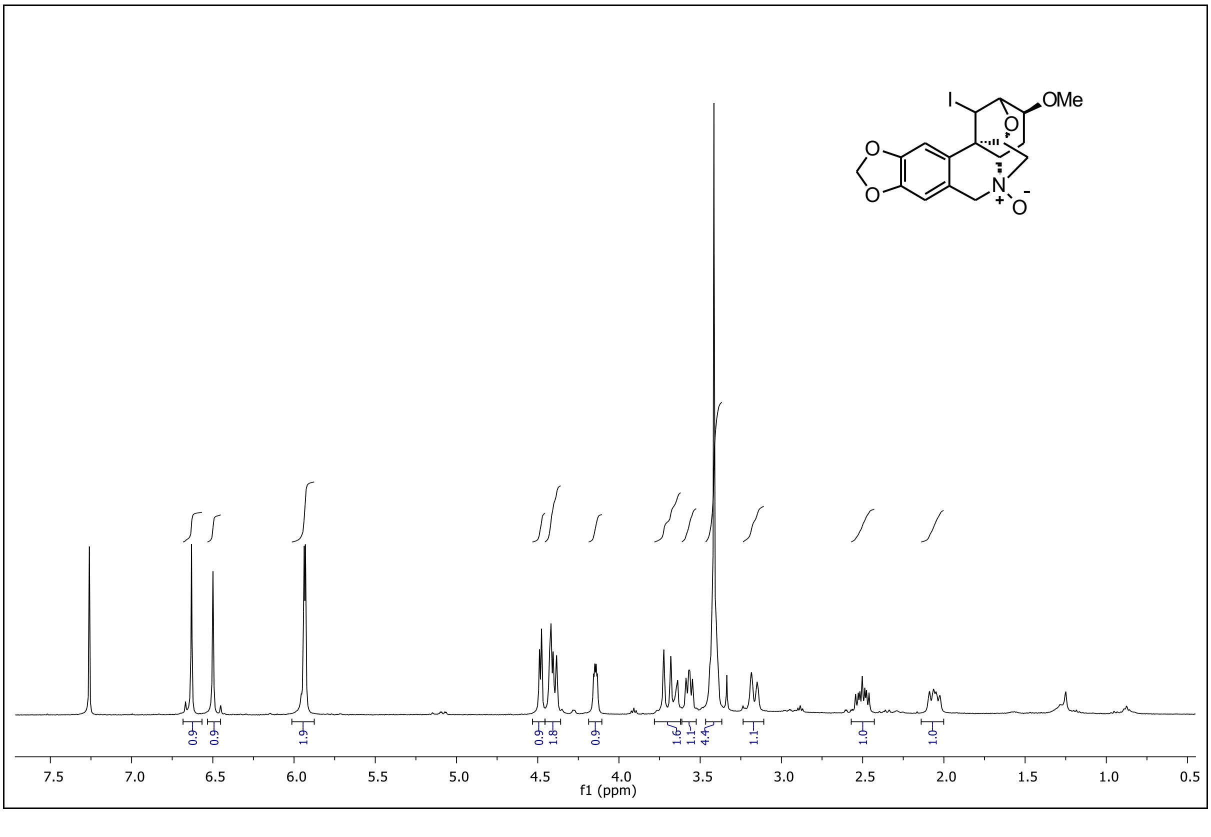

4: ^{1}H NMR (400 MHz, $CDCl_3$)

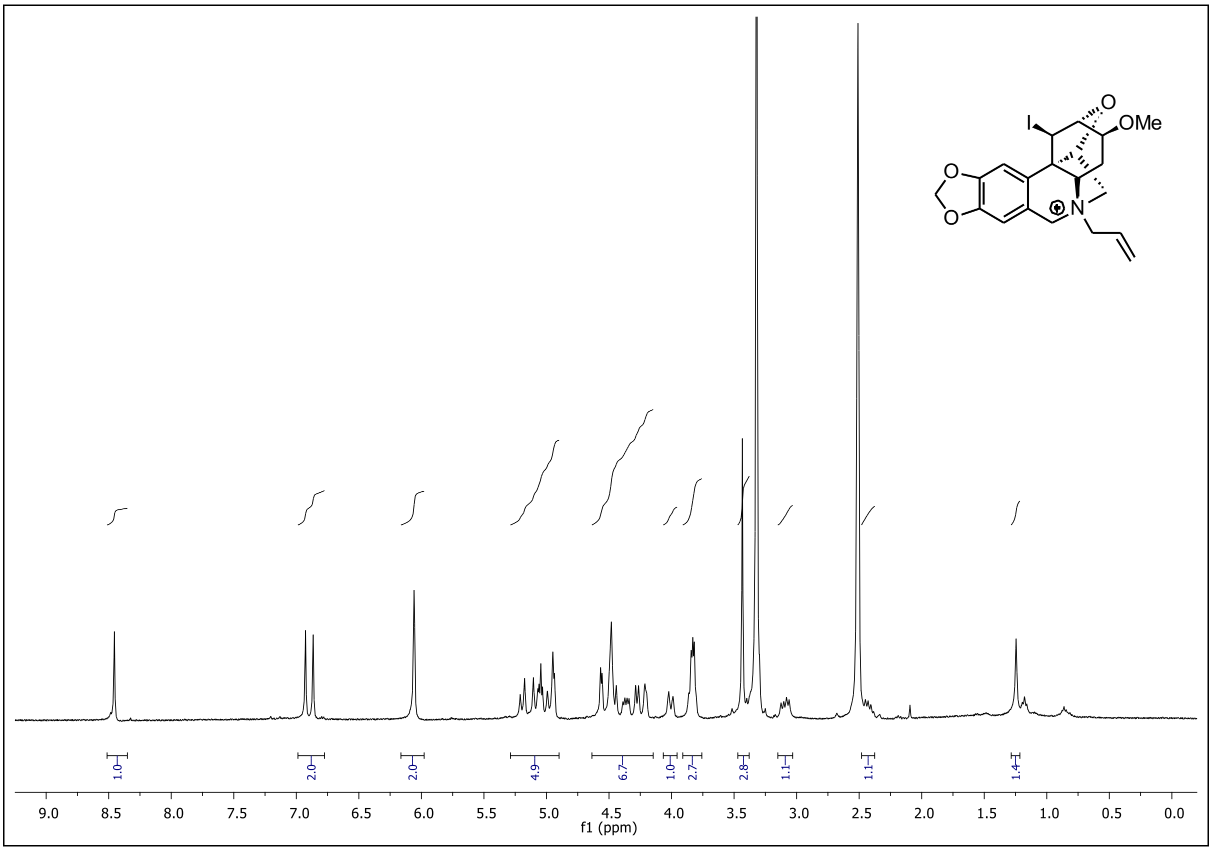

4: ^{13}C NMR (100 MHz, $CDCl_3$)

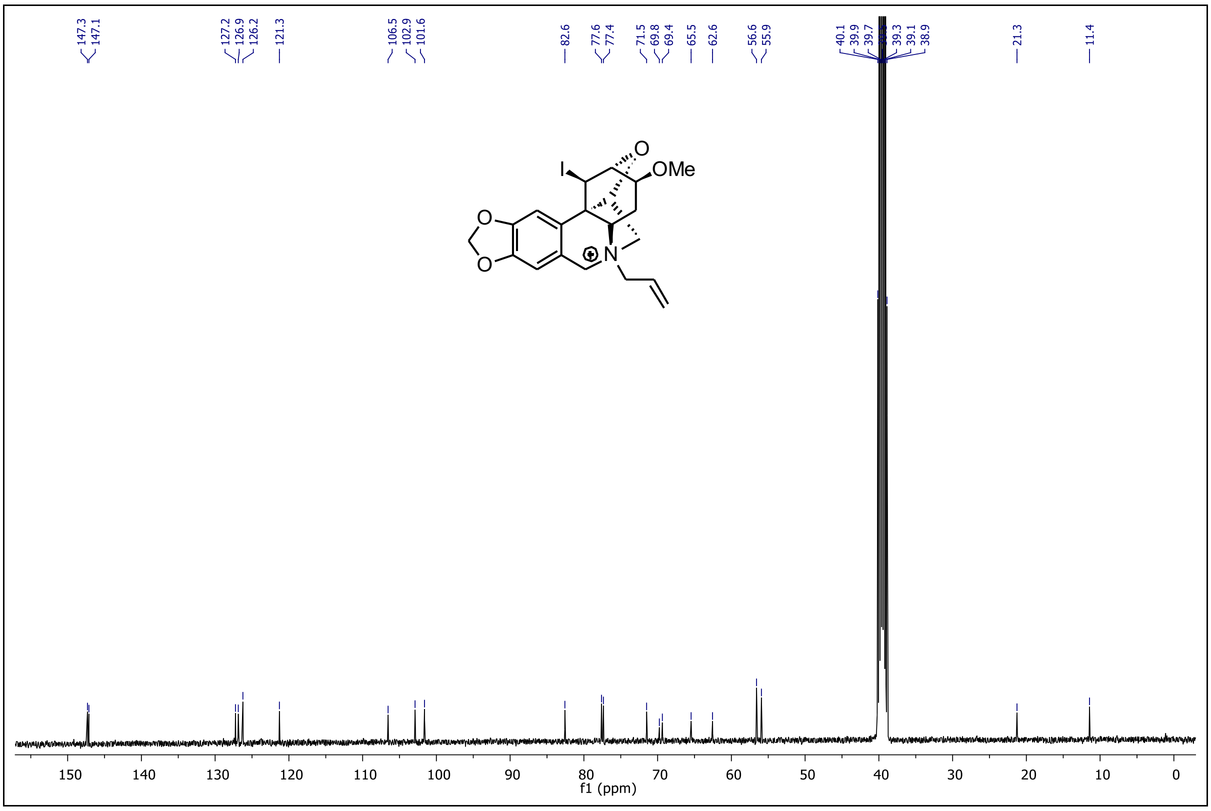

5: ^{1}H NMR (400 MHz, $CDCl_3$)

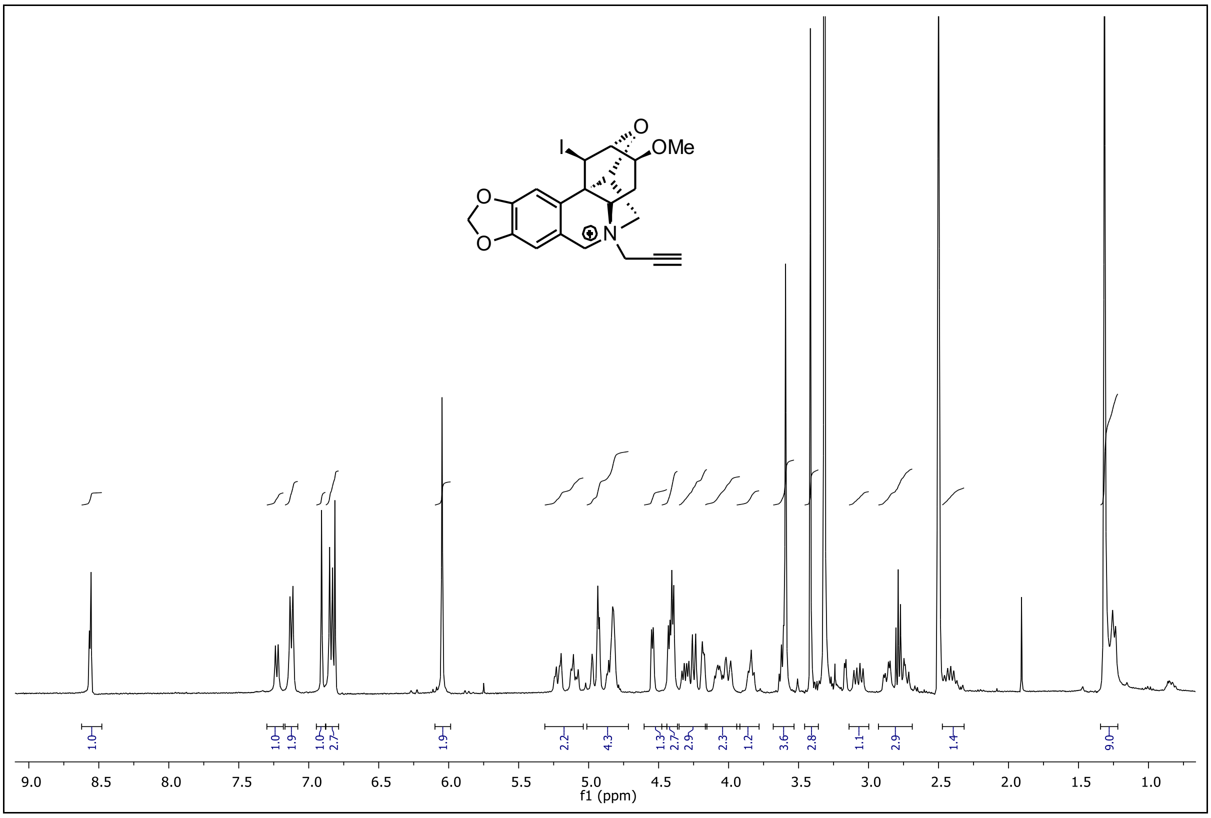

5: ^{13}C NMR (100 MHz, $CDCl_3$)

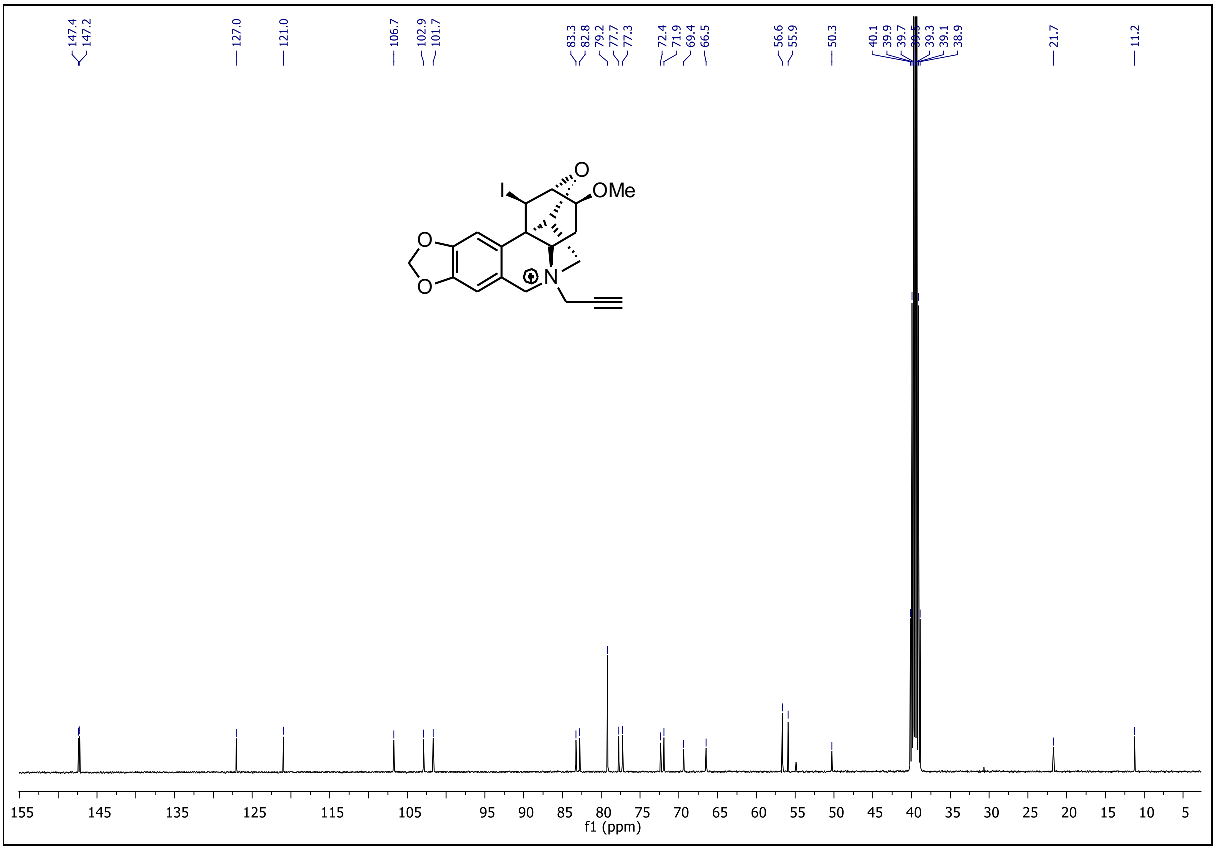

6: ^{1}H NMR (400 MHz, $CDCl_3$)

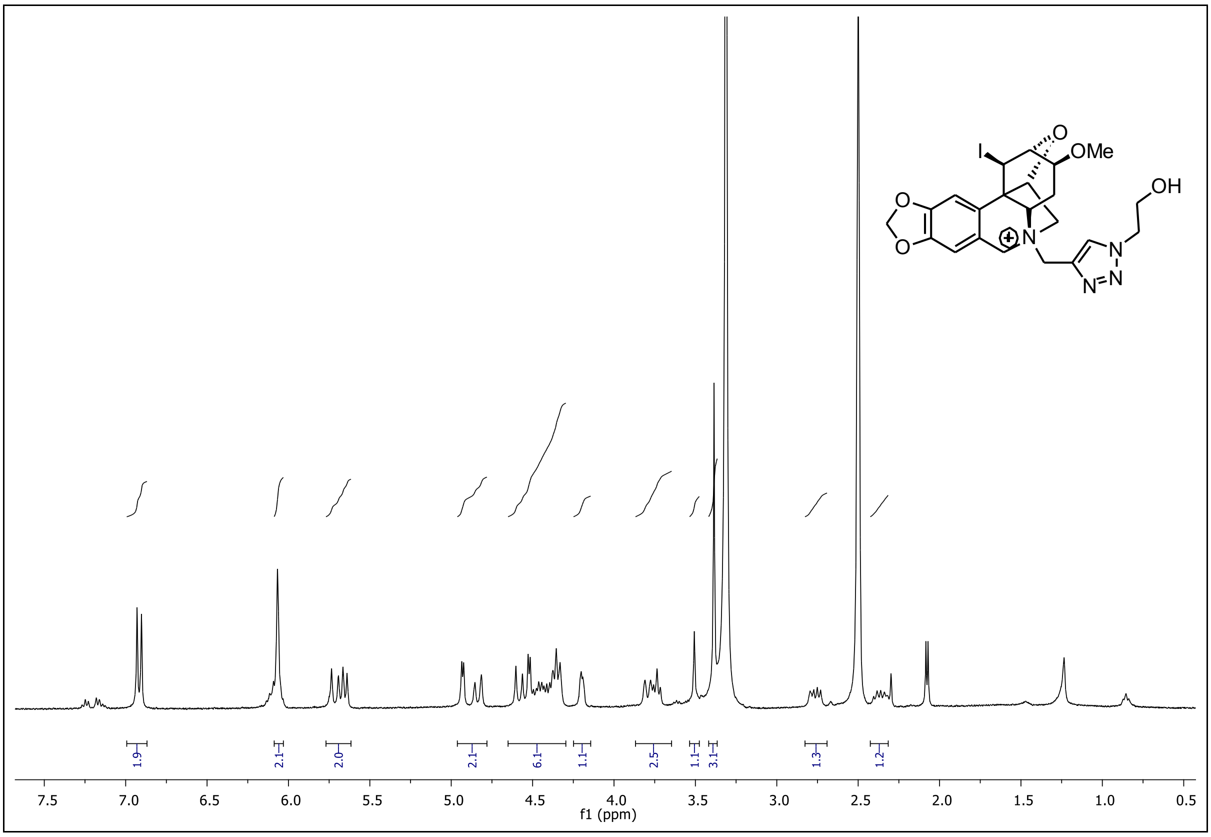

6: ^{13}C NMR (100 MHz, $CDCl_3$)

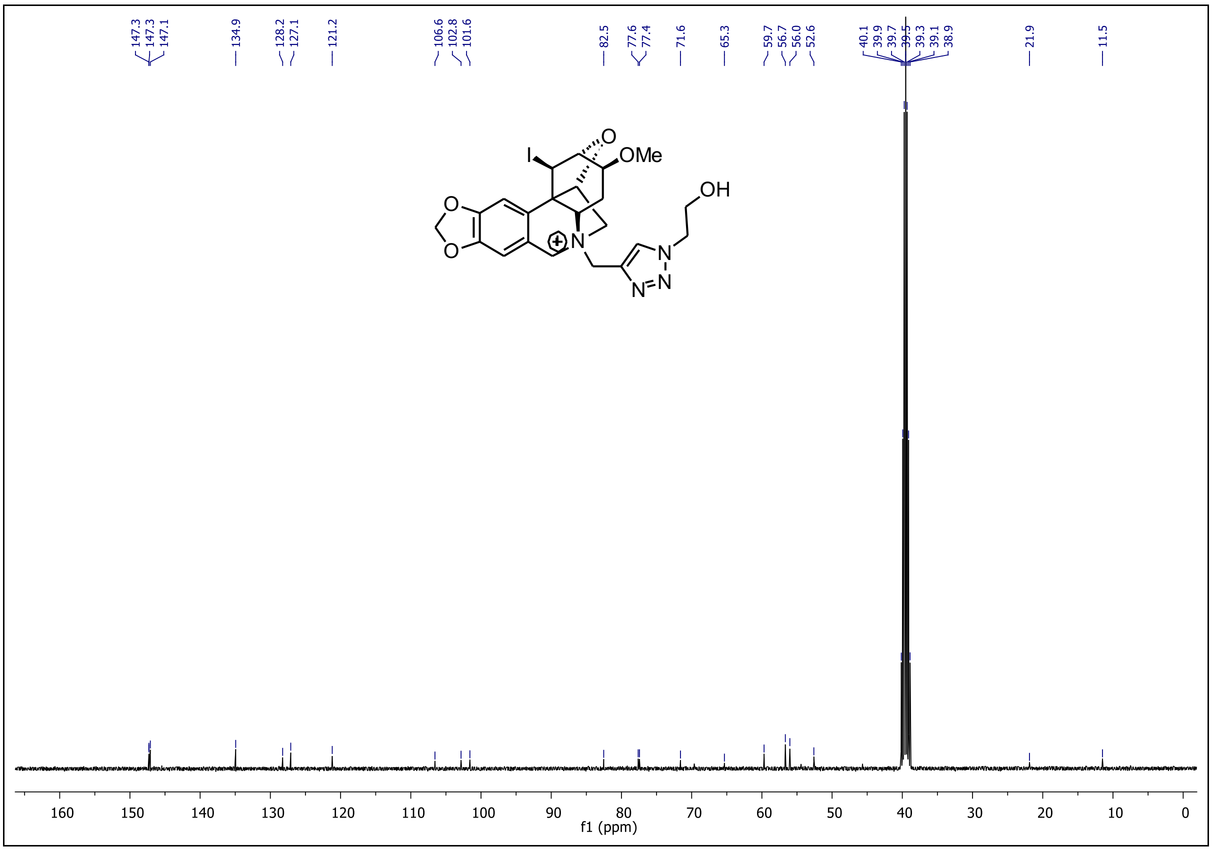

7: ^{1}H NMR (400 MHz, $CDCl_3$)

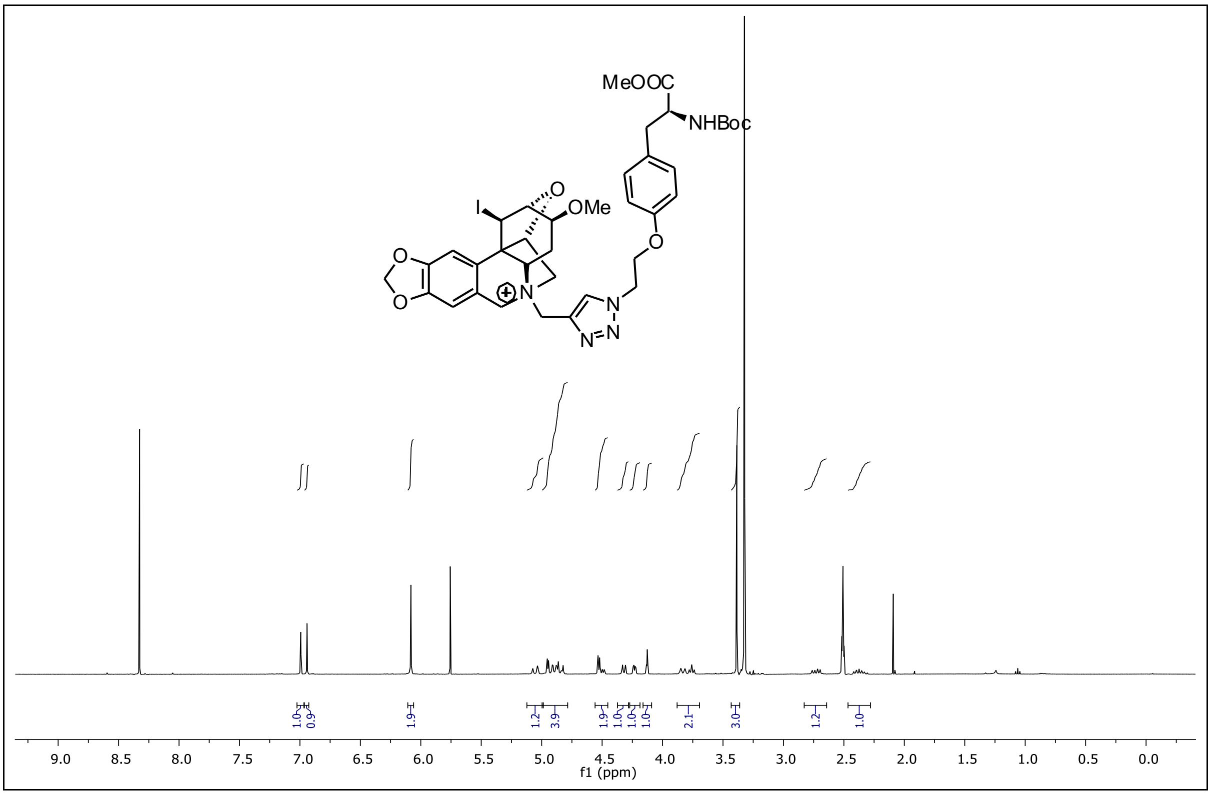

7: ^{13}C NMR (100 MHz, $CDCl_3$)

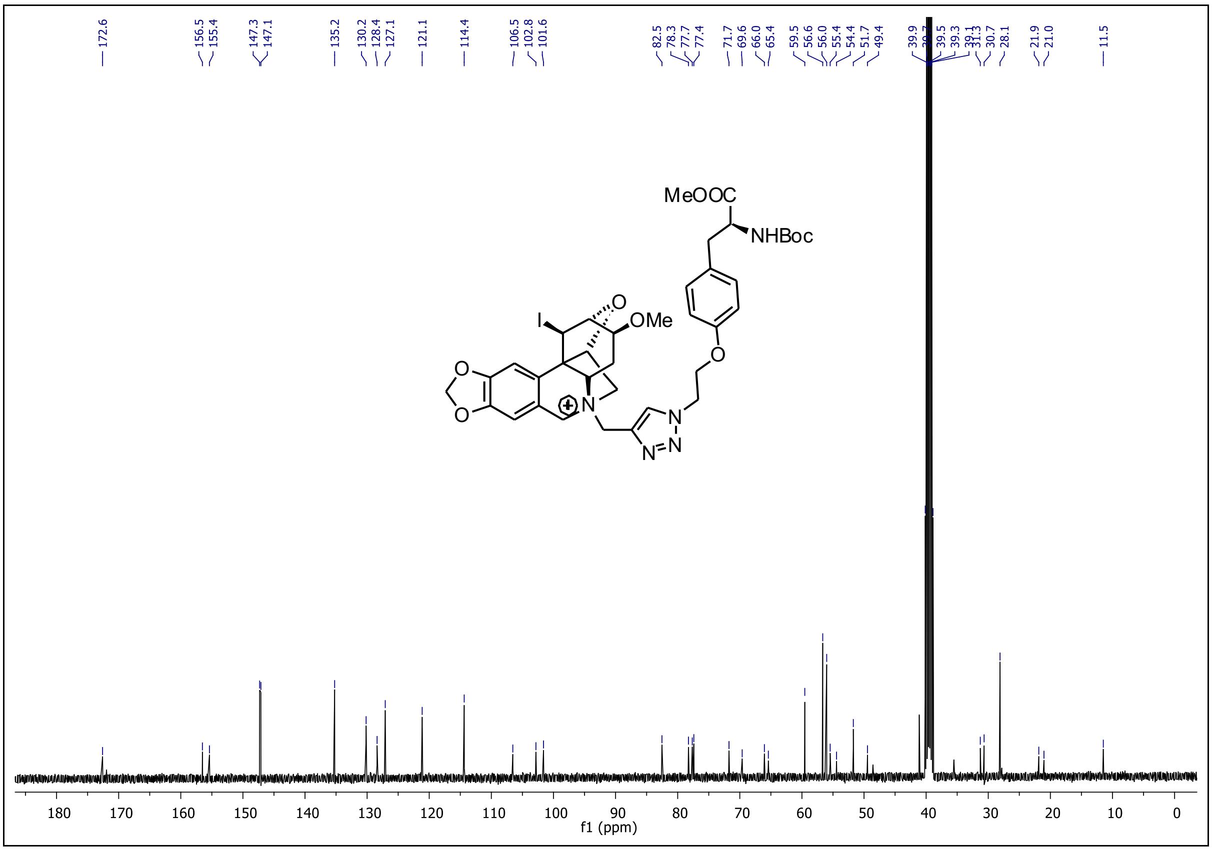

9: ^{1}H NMR (400 MHz, $CDCl_3$)

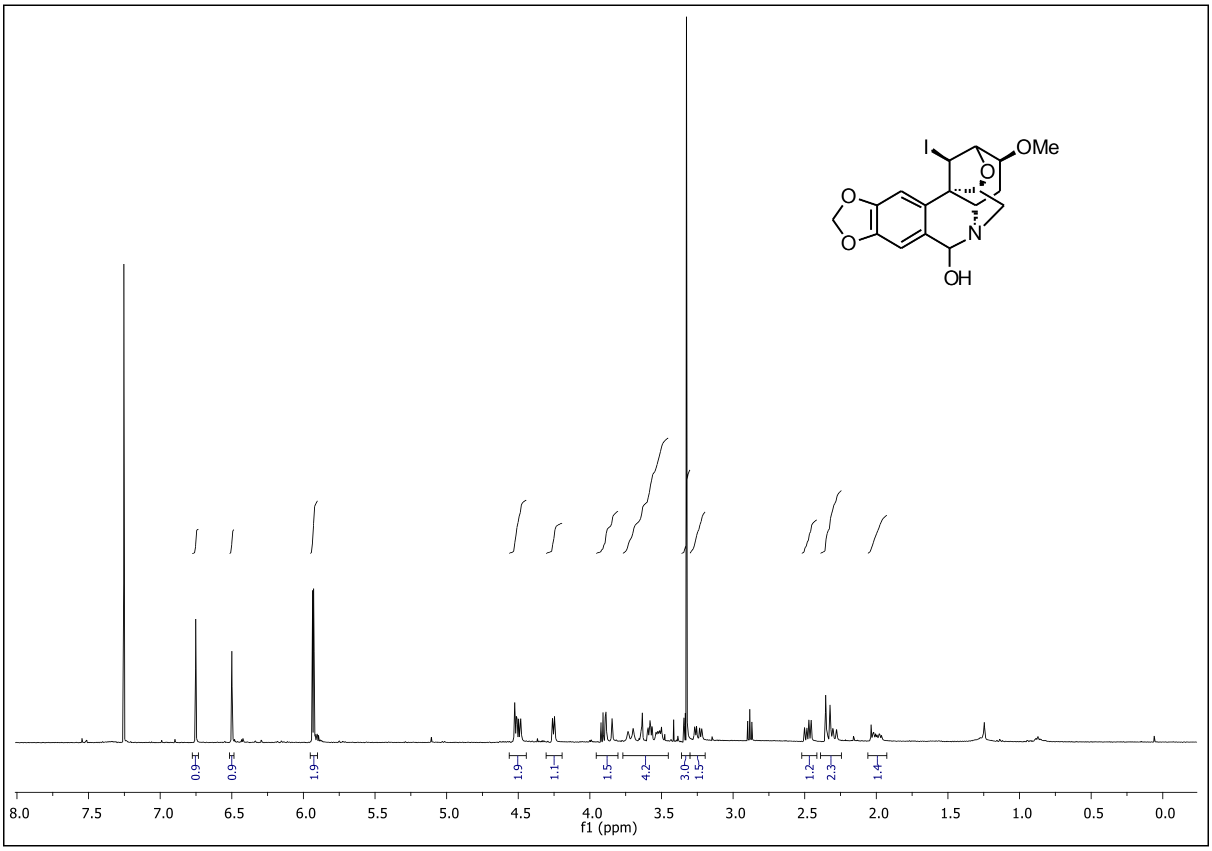

Supplement: Supplementary file 1 [file molecules-23-00255-s001.pdf]
